# Semantically enabling clinical decision support recommendations

**DOI:** 10.1186/s13326-023-00285-9

**Published:** 2023-07-18

**Authors:** Oshani Seneviratne, Amar K. Das, Shruthi Chari, Nkechinyere N. Agu, Sabbir M. Rashid, Jamie McCusker, Jade S. Franklin, Miao Qi, Kristin P. Bennett, Ching-Hua Chen, James A. Hendler, Deborah L. McGuinness

**Affiliations:** 1 Rensselaer Polytechnic Institute, 110 8th St, 12180 Troy, NY USA; 2 IBM Research, Cambridge, USA; 3 IBM Research, Yorktown Heights, USA

**Keywords:** Disease Characterization, Knowledge Representation, Guideline Modeling, Data Integration

## Abstract

**Background:**

Clinical decision support systems have been widely deployed to guide healthcare decisions on patient diagnosis, treatment choices, and patient management through evidence-based recommendations. These recommendations are typically derived from clinical practice guidelines created by clinical specialties or healthcare organizations. Although there have been many different technical approaches to encoding guideline recommendations into decision support systems, much of the previous work has not focused on enabling system generated recommendations through the formalization of changes in a guideline, the provenance of a recommendation, and applicability of the evidence. Prior work indicates that healthcare providers may not find that guideline-derived recommendations always meet their needs for reasons such as lack of relevance, transparency, time pressure, and applicability to their clinical practice.

**Results:**

We introduce several semantic techniques that model diseases based on clinical practice guidelines, provenance of the guidelines, and the study cohorts they are based on to enhance the capabilities of clinical decision support systems. We have explored ways to enable clinical decision support systems with semantic technologies that can represent and link to details in related items from the scientific literature and quickly adapt to changing information from the guidelines, identifying gaps, and supporting personalized explanations. Previous semantics-driven clinical decision systems have limited support in all these aspects, and we present the ontologies and semantic web based software tools in three distinct areas that are unified using a standard set of ontologies and a custom-built knowledge graph framework:

(i) guideline modeling to characterize diseases,

(ii) guideline provenance to attach evidence to treatment decisions from authoritative sources, and

(iii) study cohort modeling to identify relevant research publications for complicated patients.

**Conclusions:**

We have enhanced existing, evidence-based knowledge by developing ontologies and software that enables clinicians to conveniently access updates to and provenance of guidelines, as well as gather additional information from research studies applicable to their patients’ unique circumstances. Our software solutions leverage many well-used existing biomedical ontologies and build upon decades of knowledge representation and reasoning work, leading to explainable results.

This is an extended version of a previously accepted workshop paper [1] at the Semantic Web solutions for large-scale biomedical data analytics 2019 workshop [2].

## Background

Clinical Practice Guideline (CPG) documents consist of a set of evidence- and expert-based recommendations for the diagnosis, treatment, and management of specific health conditions. CPGs are usually initially published in textual form and are later translated by domain experts and information technology specialists into rules within a Clinical Decision Support (CDS) system. These guidelines are widely considered by the healthcare community as a standard source of clinical knowledge. For this reason, there have been ongoing research efforts to model the knowledge within CPGs so it can be executed within computer-based CDS systems to provide point-of-care recommendations. Example guideline models developed over the past 30 years include Proforma [3], EON [4], PRODIGY [5], Asbru [6], GLEE [7], GLARE [8], SAGE [9], each of which supports different types of reasoning capabilities. To resolve the heterogeneity of knowledge representations across models, GLIF [10] was developed as an interchange language for guideline knowledge. Furthermore, OpenClinical.org [11] has served as an online clearinghouse of models and tools. Recent efforts in guideline modeling include the ATHENA project that was generalized from hypertension to pain management and other health conditions [12] and DESIREE for breast cancer [13]. Through the encoding and implementation process of CPGs, the clinical relevance, study provenance, and evidence specificity of a CPG recommendation are often not captured or conveyed when a rule is actuated. Healthcare providers may be less inclined to follow the recommendations without an understanding of their source or their applicability [14–17]. Provider acceptance and use of CPG recommendations depend on several other factors. A healthcare provider must

(i) view the recommendation as relevant to his or her patient’s clinical situation [16–18],
(ii) accept that the research publication or publications supporting the recommendation are of high enough quality to use under time pressure [19], and
(iii) understand that the study population is similar enough to his or her patient or patient population to be applicable [20–23].

Decisions made by healthcare providers often involve complex, inferential processes often guided by alerts. The alerts in CDS systems that actuate recommendations derived from CPGs may be based on a rigid decision tree, accessing only specific and limited patient information, which may not include the relevant information about the patient needed for personalized care. Some of the difficulties associated with implementing and following CPGs in medical practice are discussed in [24]. Another unfortunate, unintended consequence of CDS systems is “alert fatigue,” due to their high false-positive rate resulting from a generic rule-based approach that does not consider personalization or contextual aspects, which can lead to providers ignoring the recommendations from the CDS system [25]. Developing a CDS system that only alerts the healthcare provider when appropriate, reduces the need for overrides, and assists with complex decision-making processes, such as providing a differential diagnosis personalized to each patient.

CPG recommendations ideally embody both the evidence and expert opinion that is the end-result of a thorough and systematic review and evaluation of the available scientific literature [26]. Then, the CDS system that applies knowledge from the CPGs should preserve the quality of evidence associated with each recommendation. For example, in the American Diabetes Association (ADA) standards of care guidelines, there is a rating on each recommendation that indicates the guideline author’s view of the scientific rigor of the supporting research publications [27]. The breadth of the biomedical literature available and the changing nature of the CPGs require providers to keep abreast of the newest additions and changes to the old guidelines more frequently than ever before. Furthermore, it can be prohibitively time-consuming for the provider to identify scientific literature relevant to a complicated patient whose needs are not addressed in CPGs. Therefore the availability of the evidence ratings and the pointers to the supporting scientific literature into the CDS systems can be beneficial for healthcare providers. With these challenges in mind, we have developed several ontologies and semantic web applications.

Specifically, we describe several solutions that can be used to answer key questions:

(i) *Can we characterize a disease based on the information available in a guideline and provide semantic links to other relevant concepts to make clinical decision support systems more explainable?* For example, a complicated disease such as breast cancer will require connections to other knowledge sources for a healthcare provider to deliver the optimal treatment option.
(ii) *Can we represent guideline provenance in a way that enables the tracking of revisions in guidelines that lead to a better understanding of the evolution of the guideline as new medical evidence comes to light?* For example, guidelines that get updated frequently, such as the diabetes standards of care clinical practice guidelines, may be too time-consuming and challenging for busy healthcare providers to process. If the changes are made available in a computable manner, the guidelines would be easily processable by healthcare providers.
(iii) *Can we represent study cohorts reported in the medical literature in a way that enables effective querying to pinpoint research publications that may be applicable for a patient and to identify gaps in guidelines?* For example, a healthcare provider might want to drill down to some studies that may be more applicable to a complicated patient (someone with multiple comorbidities) from an under-represented ethnic group rather than following the procedures for diagnosis and treatment as available in the guidelines, which may be more suitable to a general patient population.

To answer the above questions, we implemented foundational semantic software technologies. The implementations are described in Section Implementation, and how they have been applied is outlined in Section Results. In each section, we demonstrate the capabilities of a semantically enabled CDS system in the following aspects:

1. Characterizing a disease based on the CPGs as well as other supporting trustworthy sources (Sections Characterizing Disease Based on Guideline Modeling and Breast Cancer Characterization).
2. Connecting a study to a guideline recommendation (Sections Modeling of Guideline Provenance and Guideline Provenance).
3. Modeling the people studied in the research publications that support a guideline (Sections Modeling of Study Cohorts in Research Studies Cited in Clinical Practice Guidelines and Representing Study Populations).

### Use Case

Diabetes is a serious health problem that affects more than 7% of adults in developed countries and up to 16% of patients with breast cancer have diabetes [28]. Individuals with diabetes are known to be at a higher risk of developing breast cancer [29]. Suppose a healthcare provider is treating a diabetic patient with multiple comorbidities, including breast cancer, or who has a propensity to develop breast cancer. This healthcare provider may be interested in understanding how to characterize the diseases their patient has, which treatment options are available amidst the voluminous amounts of literature available to them each year through changes to the CPGs, and the evidence for and against those treatments, especially if their patient is from an under-represented community in clinical trials. Therefore, while CPGs are considered the standard for patient management, healthcare providers’ acceptance of CPGs depends on their relevance to their patient’s unique clinical situations. Furthermore, the healthcare provider’s acceptance of the CPG may rely on the published studies backing the evidence for the CPG. Therefore a CDS should cover all decision points when treating a patient and determine which recommendation(s) were relevant at a decision point and if a physician followed them.

Our CDS software implementation uses an example of a comorbid patient with diabetes and a breast cancer patient from an under-represented community. We leverage semantic technologies to characterize diseases such as breast cancer (Section Characterizing Disease Based on Guideline Modeling) as this is a complicated disease that requires a lot of rich knowledge representation. We model guideline provenance to track any changes to CPGs that change every year, such as the ADA (Section Modeling of Guideline Provenance) as it relates to breast cancer. We also model the study cohort aspects of research publications listed in the guidelines to understand how representative the guideline is to the patient (Section Modeling of Study Cohorts in Research Studies Cited in Clinical Practice Guidelines). We present state-of-the-art existing semantics-based solutions for each task that augment CDS systems followed by our methodology in Section Implementation and outline the capabilities of the software developed in Section Results.

## Implementation

### Characterizing Disease Based on Guideline Modeling

With new advances in medical research, guidelines continue to increase in complexity. Furthermore, as the data streams for diagnosing and treating diseases become complicated, providers may have to consult many different sources and use knowledge from clinical trials and literature to decide on alternative treatment options, which can take a great deal of the providers’ and the patients’ time. We believe that semantic-based techniques can overcome the challenges of perusing information available in many sources. Consulting a good number of sources that might provide alternative treatments with high-quality evidence enables providing up-to-date provenance-preserving knowledge that characterizes a patient’s disease or identifies treatment options accurately and efficiently. As an example, the American Joint Committee on Cancer (AJCC) publishes the official cancer staging system in the United States of America. As is common in many disease characterization tasks, the staging of cancer is an evolving science; When new information becomes available about etiology and various methods of diagnosis and treatment, the classification and staging of cancer evolve [30]. Since the inception of the cancer staging manual in 1977, there have been eight editions. The AJCC $8^{th}$ Edition [31] makes a substantial effort to incorporate biological and molecular markers to create a more personalized approach using pathological prognostic staging. The previous breast cancer staging guidelines (i.e., AJCC $7^{th}$ edition [32] and earlier) only considered anatomical features: the size of the tumor, the number of local lymph nodes affected, and whether the cancer had metastasized. Additionally considered in the new staging guidelines are biomarkers, including human epidermal growth factor receptor 2, estrogen and progesterone receptor statuses, and tumor grade.

#### Related Work in Cancer Characterization

Initial work related to an ontology that captured cancer staging information is available in Massicano et al. [33] where they developed computable guidelines for the Union for International Cancer Control (UICC) guideline on cancer staging. Boeker et al. [34] have also created an ontology for the same Tumor, Node, and Metastasis (TNM) based tumor classification guidelines put forth by the UICC and the AJCC $6^{th}$ edition [35] in which they focus on other, specific tumors. Beierle et al. [36] have developed the Ontology for Cancer Therapy Application (OCTA) that provides general knowledge about active ingredients and therapy regimens with an emphasis on co-medication and drug interactions to be used in a CDS system. In our work, we have learned from these previous efforts but focus primarily on the challenges providers face if the CDS does not use the most updated criteria from CPGs to characterize diseases. The most significant difference between the previous ontologies and our cancer staging ontology is the inclusion of additional biomarkers, as per the AJCC $8^{th}$ edition staging criteria, which were not available in the previous staging editions. The biomarkers used in the new edition significantly increased the complexity of the criteria required to stage a tumor. Another difference is that, as part of our semantic web tools, we utilize automated mechanisms (i.e., scripts) to extract the staging criteria from the guidelines automatically and construct the Web Ontology Language (OWL) axioms from those. Thus our method is scalable in the face of rapidly changing guideline information.

#### Methodology for Cancer Staging Characterization

Our workflow for characterizing breast cancer according to the authoritative guidelines includes the following steps. We first translated the staging criteria available in the AJCC $7^{th}$ and $8^{th}$ edition manuals into structured mappings. For this process, we extracted 19 criteria from the AJCC $7^{th}$ edition and 407 criteria for clinical prognostic stage grouping from the AJCC $8^{th}$ edition, that include different combinations of cancer staging parameters. Then, based on the mappings, we generated the OWL axioms for the staging criteria. We then integrated the recommended treatment and monitoring options available in the National Comprehensive Cancer Network (NCCN) guidelines [37], which provide information about the suitable treatment and monitoring options based on the stage. The process is outlined with all the details in [38].

**Fig. 1:**
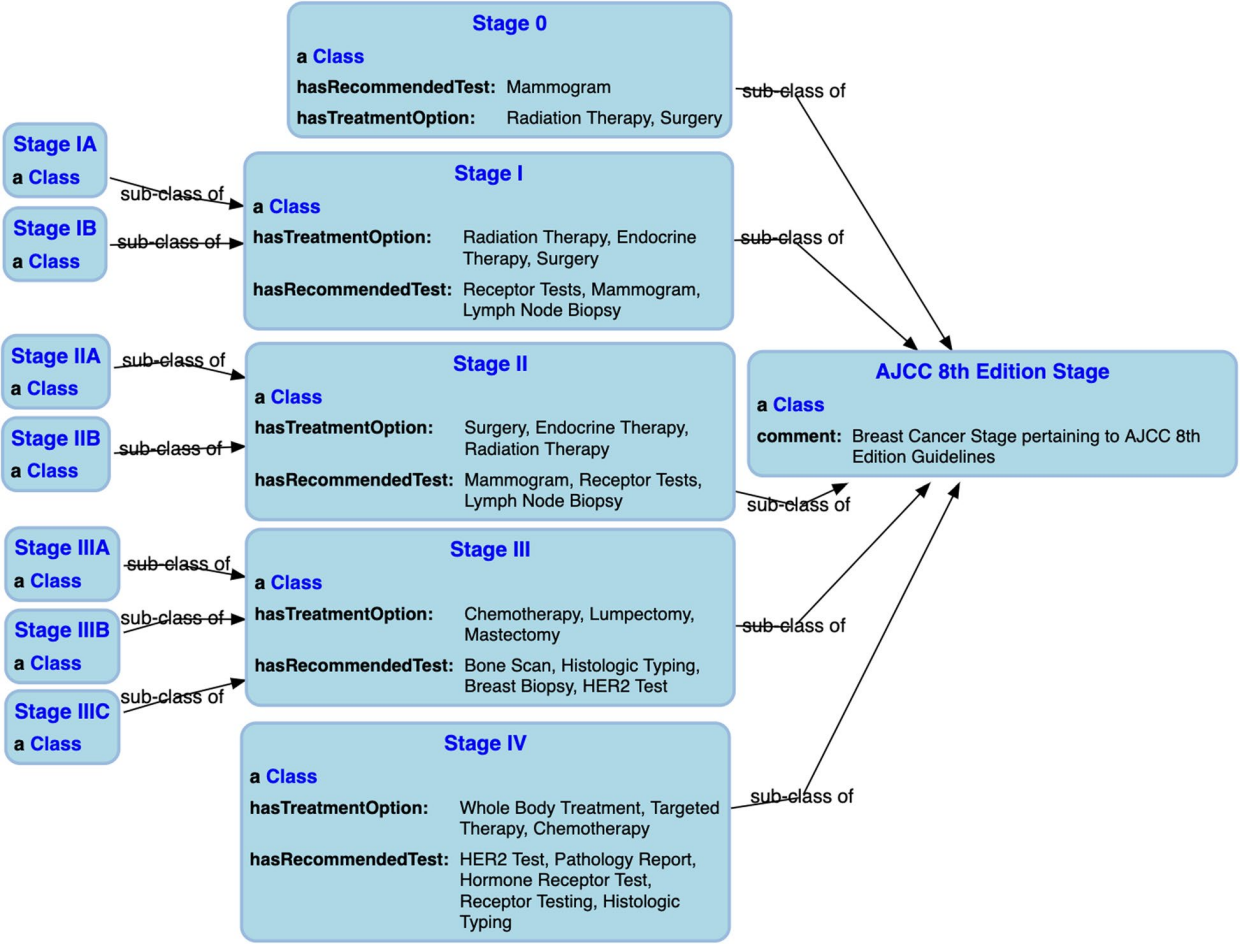
Breast cancer stage hierarchy classes as per the authoritative cancer staging CPGs

As can be seen in Fig. 1, which depicts the AJCC $8^{th}$ staging edition staging class hierarchy, each stage class includes the properties hasRecommendedTest, hasTreatmentOption, and subClassOf assertions where applicable. We added the comments to better describe the concepts in the ontology, based on the descriptions available in the medical literature and to support explanations. In addition to including classes for all cancer stages for the respective guideline, we also mapped the breast cancer terms to community-accepted terms from the National Cancer Institute Thesaurus (NCIT) [39] and incorporated recommended tests and treatment plans from the openly reusable Clinical Interpretations of Variants in Cancer (CIViC) [40] data, to provide additional stage-specific recommendations.

### Modeling of Guideline Provenance

Recording the provenance of guidelines helps address the challenging task of systematically updating a system when new guidelines or medical literature are published. Providing provenance of guidelines implicitly or explicitly being used by CDS systems can enhance the trust of healthcare practitioners in these systems by increasing the transparency of the CDS system. Provenance can also aid in resolving ambiguity and conflicts between various guideline sources.

#### Related Work in Capturing Provenance

Several general-purpose provenance ontologies currently exist, such as the Provenance Ontology (PROV-O) [41], the World Wide Web Consortium (W3C) recommendation for encoding provenance that provides classes and properties to capture generic provenance terms in various domains. The Dublin Core metadata Terms (DCT) [42] is a lightweight vocabulary that aids in the description of resources and provides a list of terms to describe metadata information for articles, figures, and tables at a higher level. The Bibliographic Ontology (BIBO) [43] can be used to provide a more detailed description of referenced articles. These provenance ontologies address the issue of modeling provenance at a more general level and do not address how to represent guideline provenance in particular. In our solution, we build on these foundational provenance ontologies to associate provenance of clinical decisions with their authoritative guideline evidence.

In the clinical domain, there have been several efforts to create ontologies that enable increased traceability, transparency, and trust in clinical data. Provenance for Healthcare and Research (ProvCaRe) [44] is an ontology designed to enhance the reproducibility of scientific research by capturing the metadata of published articles. Their ontology focuses on the details of the scientific study, including the design description, the data collection methods, analysis of the data, and overall research methodology. Provenance Context Entity [45] is a scalable provenance tracking for scientific Resource Description Framework (RDF) Data that creates provenance-aware RDF triples using the provenance context notion. Provenance in a CDS system that attempts to trace all the system’s execution steps to explain, on the patient level, the final results generated by the system is investigated in [46]. There is also extensive research in provenance in distributed healthcare management, which aims to ensure an efficient healthcare data exchange [47–49]. Several works have proposed an ontology-based solution to provide appropriate guidance to patients suffering from chronic illnesses such as diabetes, including an ontological modeling and reasoning of CPG contents [50], and the OntoDiabetic system that can assess the risk factors and provide appropriate treatment [51].

The ontology we developed is different from prior approaches in that we encode the information present in published CPGs into computable knowledge, such as rules, as well as the evidence sentences from the CPG directly. In our approach, we identified the various pieces of information about the source of a recommendation that a healthcare provider needs to make clinical decisions.

#### Methodology for Capturing Provenance in CPGs

We developed an ontology called Guideline Provenance Ontology (G-Prov) [52] with three key clinical competency questions in mind:

(i) Where does this treatment suggestion come from?
(ii) Which research publications support the recommendation?
(iii) How recent is this recommendation?

We modeled the G-Prov ontology with the above questions in mind to capture provenance metadata at different levels of granularity. The G-Prov ontology enables accurate encoding of the source of the reasoning rules that CDS systems rely on to generate diagnosis and treatment suggestions. For instance, G-Prov can be used to annotate rules with citations found in evidence sentences and other sources of knowledge, such as figures and tables.

When developing the G-Prov ontology, we reused several well-used existing ontologies, including the W3C PROV-O [41], DCT [42], BIBO [43], Semanticscience Integrated Ontology (SIO) [53], Disease Ontology (DOID) [54], and NCIT [39]. We also utilized our Study Cohort Ontology (SCO) [55], which is detailed further in Section Modeling of Study Cohorts in Research Studies Cited in Clinical Practice Guidelines.

The G-Prov ontology is comprised of terms broadly belonging to three classes.

(i) *Metadata descriptions* using existing ontologies such as PROV-O, DCT, and BIBO
(ii) *Guideline specific* terms that associate rules to their guideline evidence we defined
(iii) *Disease specific* classes and properties from DO, and NCIT

**Fig. 2:**
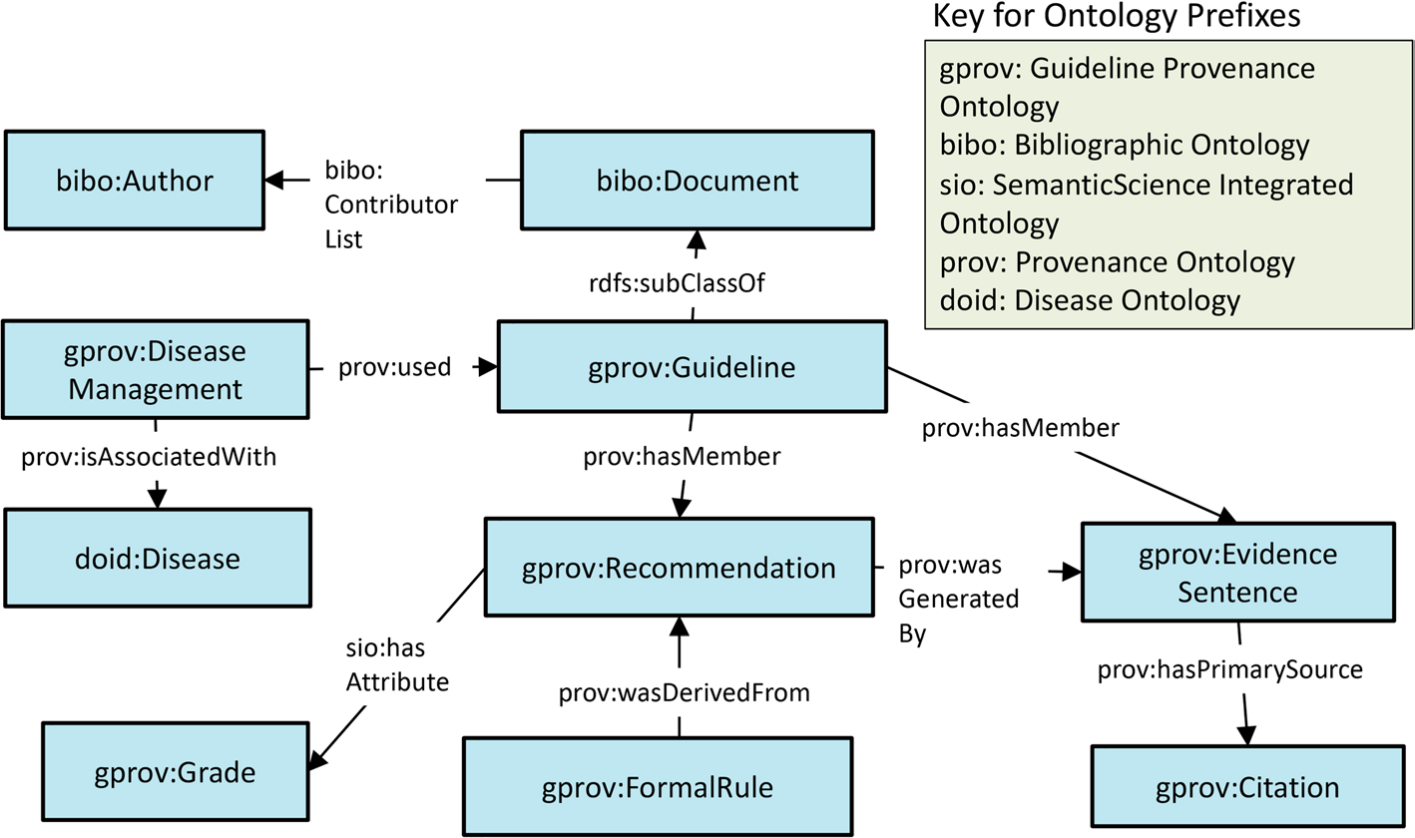
Conceptual Model of the G-Prov Ontology

As can be seen in Fig. 2 that depicts the higher-level conceptual model of G-Prov, gprov:Citation represent citations within CPGs, and are linked to their reference information using prov:hasPrimarySource. Further, every published document has at least one author, and, to capture the list of all the authors in a publication, we use the class bibo:Author, which contains one or more authors. To annotate treatment rules with G-Prov we introduced the gprov:FormalRule class. Guideline recommendations (modeled as gprov:Recommendation) contain these rules, and we use the prov:wasDerivedFrom property to link the two classes. We also link the rules directly to the guideline at a more abstract level.

Since CPGs focus on a specific health condition, we link the guideline class to the health condition it addresses using gprov:DiseaseManagement via the prov:used property. For example, the ADA CPG addresses diabetes, therefore gprov:DiseaseManagement corresponds to *diabetes* and the prov:used property corresponds to the ADA CPG. The gprov:DiseaseManagement class links to at least one specific disease type (DOID:Disease). Additionally, some guidelines carry a measure of the quality of each recommendation, and we capture this information using gprov:Grade. One or more evidence sentences, modeled as gprov:EvidenceSentence, provides the scientific rationale or healthcare provider consensus for the recommendations within the guideline.

### Modeling of Study Cohorts in Research Studies Cited in Clinical Practice Guidelines

The results of Randomized Clinical Trial (RCT), and other types of clinical research publications, provide the basis for the recommendations within CPGs. However, a provider may be interested in knowing which research publications form the basis of a particular recommendation and whether their patient or patient population is similar to the study cohort(s). Achieving this is not a simple task because the published population description varies significantly across research publications. Furthermore, a single patient may differ on multiple attributes from those in the study cohort, and comparing similarity or dissimilarity across these dimensions can be challenging. Therefore, healthcare providers need a robust representation of research publications and cohorts to evaluate and visualize cohort similarity. We have developed an ontology, the Study Cohort Ontology (SCO), to meet this need.

#### Related Work in Representing Study Cohorts

In terms of prior research in this space, the Ontology of Clinical Research (OCRe) [56] is a widely cited study design ontology used to model the study lifecycle and addresses similar goals to our work. They adopt an eligibility rule grammar and ontology annotation approach [57] for modeling study eligibility criteria to enable matching a study’s phenotype against patient data. Although their ontological model captures statistical measures, their modeling is not as intuitive and does not seem to leverage the power of OWL math constructs to the fullest. We also found that most RCT ontologies, e.g., Clinical Trials Ontology - Neuro Degenerative Diseases (CTO-NDD) [58], are domain-specific and not directly reusable for a population modeling scenario. Other ontologies, such as the EPOCH ontology [59], can be used to track patients through their RCT visits and had class hierarchies that were insufficient to represent the types of publication cited in the ADA guidelines.

In particular, RCT matching has been attempted multiple times, mainly as a natural language processing problem, including a knowledge representation approach that borrows from Systemized Nomenclature of Medicine - Clinical Terms (SNOMED-CT) to improve the quality of the cohort selection process for RCTs [60]. However, the focus of their effort was mainly on efficient knowledge representation of patient data and the formulation of the study eligibility criteria as SPARQL Protocol And RDF Query Language (SPARQL)^1^ queries on the patient schema. We tackle the converse problem of identifying research publications that apply to a clinical population, based on the study populations reported. Furthermore, Liu et al. detail an approach to creating precision cohorts [61]. Their emphasis is on learning a distance metric that best suits the patient population, but they do not provide a quantified similarity score.

Our ontology and associated applications differ from these general-purpose, study component modeling efforts and patient matching efforts in that, through our ontology, we enable the declarative representation of study populations represented in research publications. Hence, we allow the performance analysis to either identify relevant research publications for patients or gaps that would prevent the adoption of research publications for specific clinical populations.

#### Methodology for Representing Study Cohorts

We developed SCO [55] to define classes and properties to describe content related to demographics, interventions, cohort statistics for each variable of a study cohort, as well as a study’s inclusion/exclusion criteria. In our modeling effort, we leveraged best practice ontologies in four categories, and we continue to support interlinking and expansion to other relevant ontologies as needed:

(i) *Upper-Level*: SIO [53]
(ii) *Statistical*: Statistical Methods Ontology (STATO) [62], and Units Ontology (UO) [63]
(iii) *Study Design*: ProvCaRe [44], OCRe [56], Human-Aware Science Ontology (HAScO) [64], and Clinical Measurement Ontology (CMO) [65]
(iv) *Medical*: NCIT [39], Children’s Health Exposure Analysis Resource (CHEAR) [66, 67], Human Health Exposure Analysis Resource (HHEAR) [68], Phenotypic Quality Ontology (PATO) [69], and DO [70]

#### Modeling Descriptive Statistics

Representing aggregations in OWL and RDF has been a long-studied research problem, and there are multiple approaches to the modeling of aggregations [71–74]. Having analyzed patterns across several population description tables, we see that the descriptive statistics model aggregations of the cohort descriptors. The descriptive statistics are often measures of central tendency or dispersion like *mean*, *median*, *mode*, *standard deviation*, *interquartile range*, and *rate*. OWL inherently supports collections through the OWL class axioms. The OWL reference guide [75] states that “Classes provide an abstraction mechanism for grouping resources with similar characteristics. Like RDF classes, every OWL class is associated with a set of individuals, called the class extension.” We leverage this feature and represent study arms as collections of study subjects, that is < instance of owl:Class > rdfs:subClassOf sio:StudySubject. We associate subject characteristics as attributes/properties of study arms and further link the descriptive statistics via reification techniques.

#### Generating Study Cohort Knowledge Graphs

We have expanded our work on study cohort modeling by automatically extracting and assembling the study cohort information from tables in a research publication to populate the SCO Knowledge Graph (KG). Study cohort tables exhibit wide variance in representation, style, and content, thus creating challenges for direct translation into a KG. We have mitigated these variance issues by designing a four-stage study cohort extraction pipeline [76] that extracts population descriptions from study cohort tables of research publications and which thereafter utilizes a heuristics and ontology-enabled algorithm to assemble these tabular components in KGs.

**Fig. 3:**
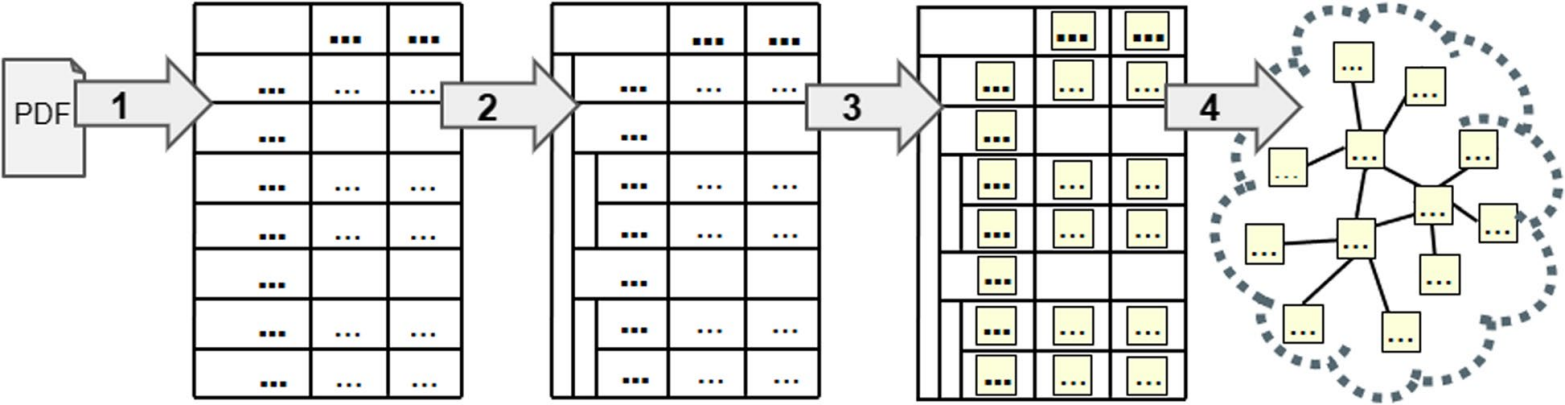
A high-level overview of the study cohort extraction pipeline. Step 1: Table text extracted from PDF. Step 2: Hierarchical structure of table identified. Step 3: Knowledge graph elements identified within the table. Step 4: Knowledge graph assembled from disparate elements

**Fig. 4:**
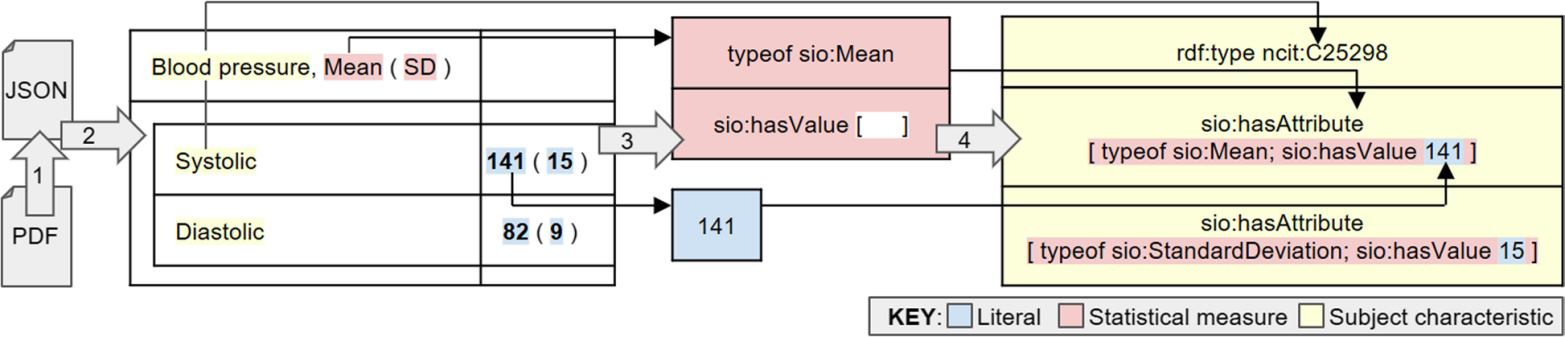
The four steps of the study cohort extraction pipeline, shown with an example snippet of a study cohort table

A high-level overview of the study cohort extraction pipeline is shown in Fig. 3, visualizing how the four stages of the pipeline work together to extract information, organize it, and produce a KG. In the first step of the pipeline, a PDF extractor tool [77] identifies the rows, columns, and cells present in a study cohort table and extracts text from within each cell. In the second step, our algorithm identifies row sub-headers within the table to convert it into a hierarchical table structure. In the third step, KG elements are identified from the text within the table, including numbers, statistical measures, and biomedical terminology. Since the terminology across the population descriptions varies from one ontology to another, we have leveraged the National Center for Biomedical Ontology (NCBO) Annotator [78] for semantic alignment. In the final step, the hierarchical structure is leveraged to assemble these elements into an SCO KG. Figure 4 shows how these four steps operate on an example of a study cohort table.

## Results

We outline three different software artifacts generated as part of this research that demonstrate the semantic enhancement of CDS systems using our approach and infrastructure.

### Breast Cancer Characterization

#### Breast Cancer Staging (BCS) Ontology

Standard terms used in the staging process were incorporated into our Cancer Staging Terms (CST) ontology [79]. Taking the different staging criteria into account, as explained above, we created ontologies for the AJCC $7^{th}$ edition, i.e., Breast Cancer Staging 7$^{th}$ Edition (BCS7) ontology [80] and the AJCC $8^{th}$ edition, i.e., Breast Cancer Staging $8^{th}$ Edition (BCS8) ontology [81]. BCS7 and BCS8 contains 22 and 31 distinct classes respectively.

**Fig. 5:**
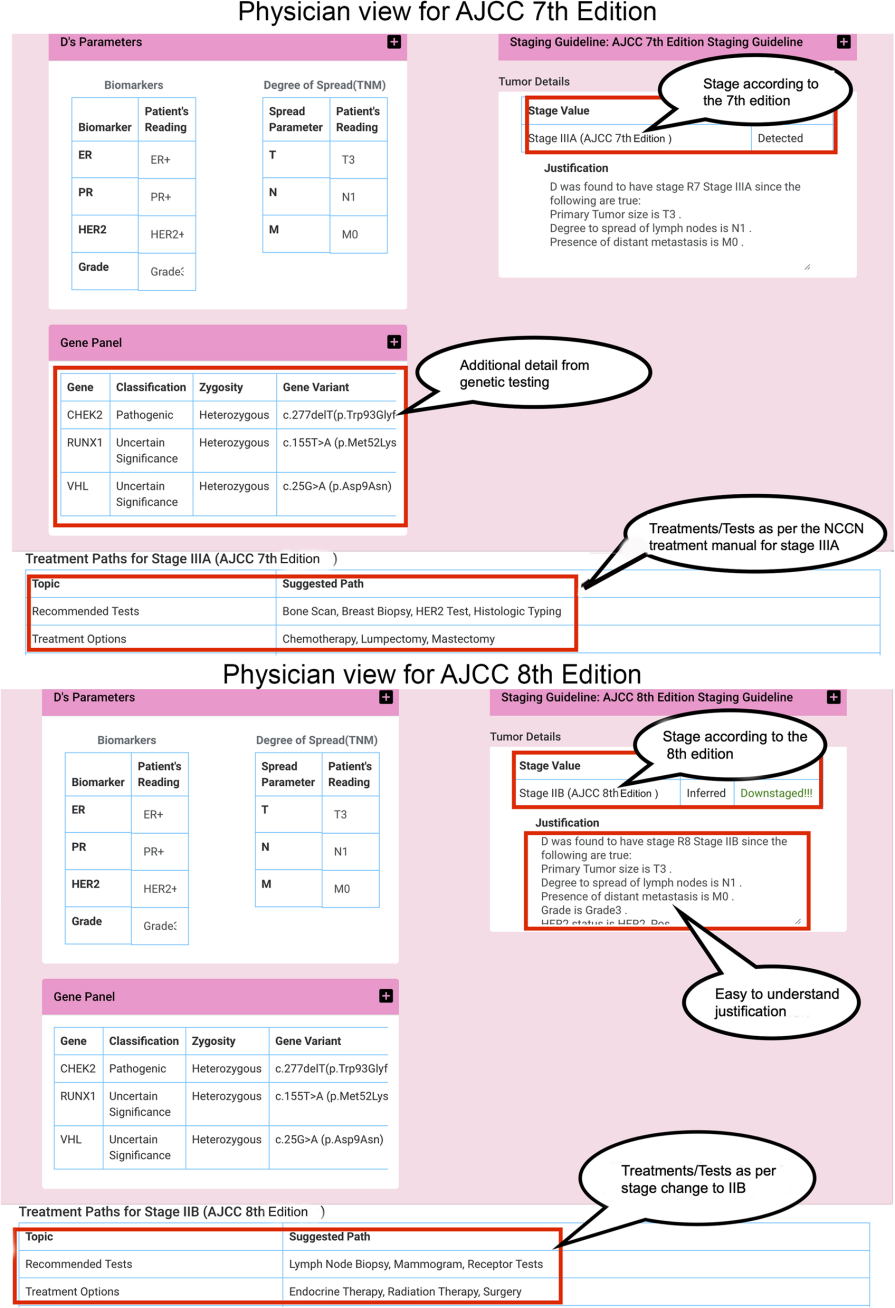
Example of downstaged breast cancer characterization

#### Inferring Cancer Stage using the BCS Ontology

We developed a deductive inference agent on the Whyis KG framework [82] to infer the stage of a tumor and the corresponding treatment and monitoring plans. The inference agent reasons over nanopublications [83] that contain sample patient data records against the CST ontology. We built SPARQL templates for different OWL reasoning profiles, as well as custom inference rules based on shape of the data contained in the patient records, to be consumed by the inference agent [38]. We obtained the patient data samples from the Surveillance, Epidemiology, and End Results (SEER) [84] datasets. We browsed the datasets using the statistical software, SEER*Stat [85] and downloaded a subset of that data to create the patient nanopublications. These datasets contained the desired features to construct the patient KG. The features extracted for the inference process include tumor stage, as per the older AJCC $6^{th}$ edition, and other relevant information, such as the demographics and the survival status of patients treated from 1980-2012.

Figure 5 shows a visualization tool that incorporates the Whyis deductive inference agent to display different treatment paths and guideline impacts on a patient in the form of an interactive report. In this visualization tool, it is possible to choose between the latest AJCC staging guidelines, i.e., AJCC $7^{th}$ and $8^{th}$ editions. Once a guideline is selected, the view dynamically loads the newly derived knowledge. The derived knowledge includes the inferred stage, whether it is up-staged/down-staged/no-change, and the explanations behind the inferred stage.

#### Software Availability

Detailed information about characterizing breast cancer staging as per the authoritative CPGs using semantic technologies is available at [86]. A similar approach can be utilized for characterizing other diseases. Furthermore, the software for the Whyis deductive inference agent is available at [87].

### Guideline Provenance

#### G-Prov Ontology

The G-Prov ontology, which is available on bioportal at [88], contains 138 classes and 317 properties. The ontology heavily reuses many concepts from existing foundational provenance ontologies, and domain-specific biomedical ontologies.

#### G-Prov Annotation of ADA CPG to Generate a KG

We demonstrate our work on annotating CDS system rules using recommendations in a CPG for type 2 diabetes using the ADA CPG, and discuss how this assists in utilizing CPGs in various medical settings. For this purpose, we used an existing ontology for diabetes management, the Diabetes Mellitus Treatment Ontology (DMTO) [89], that contains several useful rules written in the Semantic Web Rule Language (SWRL) [90]. DMTO is an ontology that provides treatment suggestions for type 2 diabetes. It uses information from several medical guidelines on diabetes, including the ADA [27], Diabetes Canada [91], and the European Association for the study of Diabetes [92]. However, DMTO lacks information on the source of each rule within the ontology. Therefore, it is difficult to evaluate the accuracy of and the evidence for each rule in DMTO. We attached provenance to the SWRL treatment rules of the DMTO with recommendations from the ADA Standards of Medical Care guideline, also known as the ADA CPG recommendation [93].

**Fig. 6:**
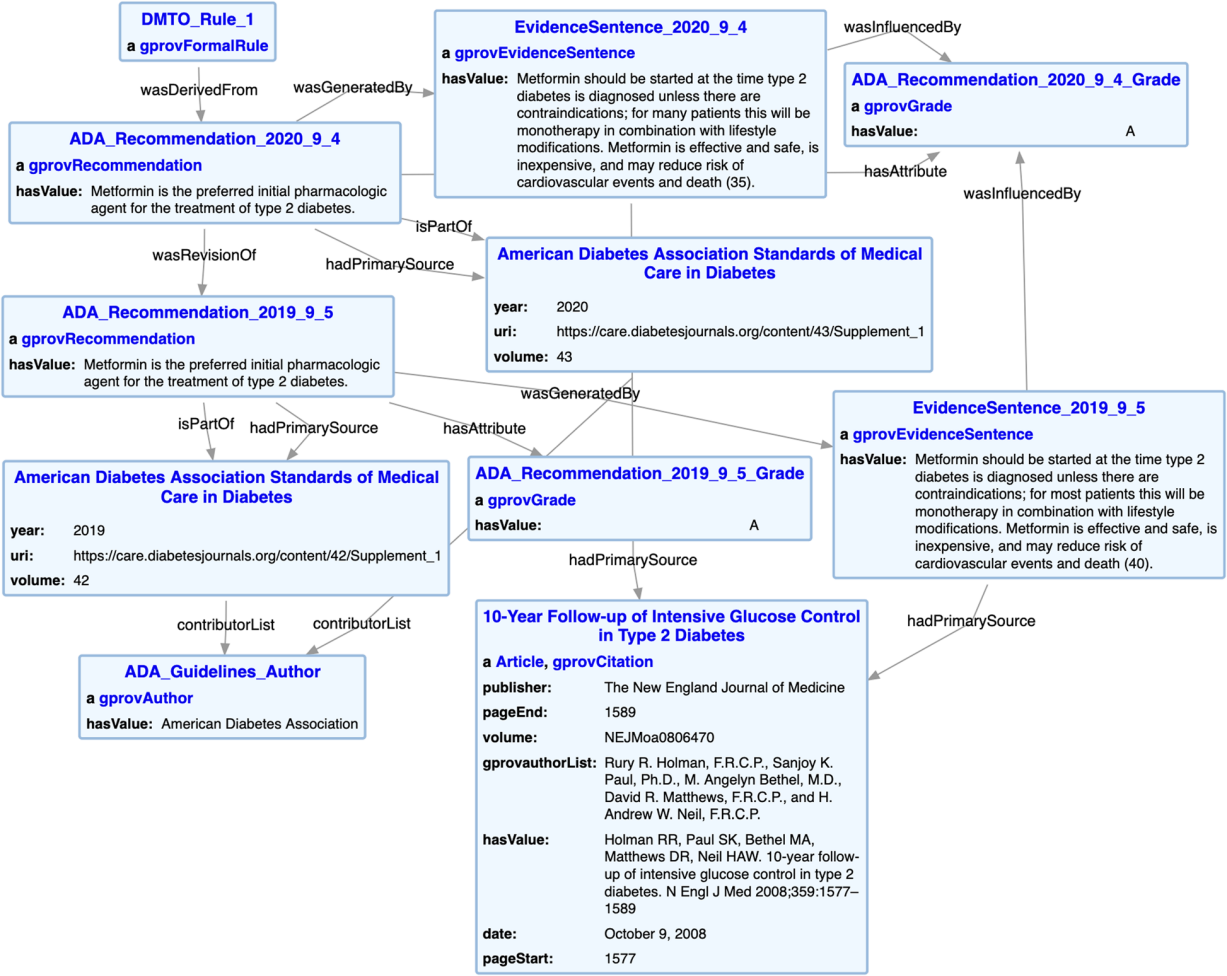
Instance diagram showing the modeling of a single recommendation within the ADA CPG using G-Prov Ontology

To annotate a treatment rule with its ADA CPG recommendation, we first did a manual pass on the CPG to understand its structure. Subsequently, we wrote a web scraping script to extract all the guideline contents into a structured data file. The content of this structured data file includes all the guideline recommendations, their grades, possible evidence sentences, and the citations within each sentence. Our initial efforts focused on two chapters from the ADA CPG, namely the “Pharmacologic Approaches to Glycemic Treatment” [94] and “Cardiovascular Disease and Risk Management” [95] chapters. To construct the KG, we created a spreadsheet to organize the extracted guideline data. Further, we manually identified applicable guideline recommendations for DMTO treatment rules and mapped them within the spreadsheet. Our medical domain expert went over these annotations to confirm both completeness and accuracy. Finally, we wrote a Semantic, Extract, Transform, and Load (SETL) script to automatically convert the content of the spreadsheet to an RDF KG [96]. Figure 6 highlights the resulting KG from modeling a selected recommendation within the ADA CPG using the G-Prov ontology.

#### Applications of G-Prov

We leveraged the G-Prov ontology and the ADA KG in an application to showcase its versatility. The application evolved out of the need for healthcare practitioners to identify the sources of treatment suggestions made by CDS systems. Our application captures three main use cases:

(i) Providing a *healthcare provider* with the provenance information of treatment suggestions made by CDS systems.
(ii) Assisting *content developers of CDS systems* in entering provenance information while creating/editing the decision rules.
(iii) Providing a *population health manager* an identification of subgroups (cohorts) of patients who may benefit from the recommendations.

**Fig. 7:**
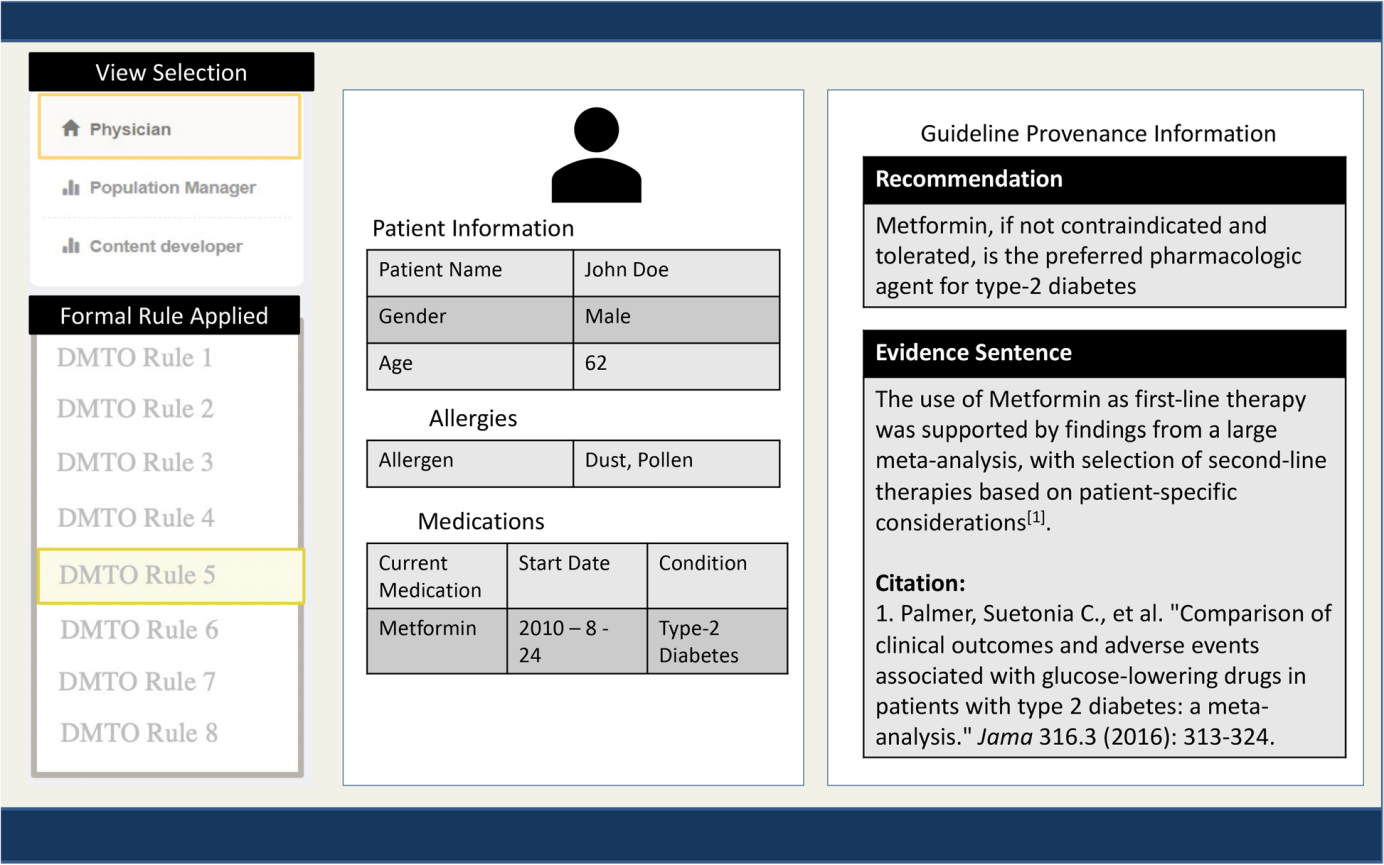
G-Prov Healthcare Providers’ View

**Fig. 8:**
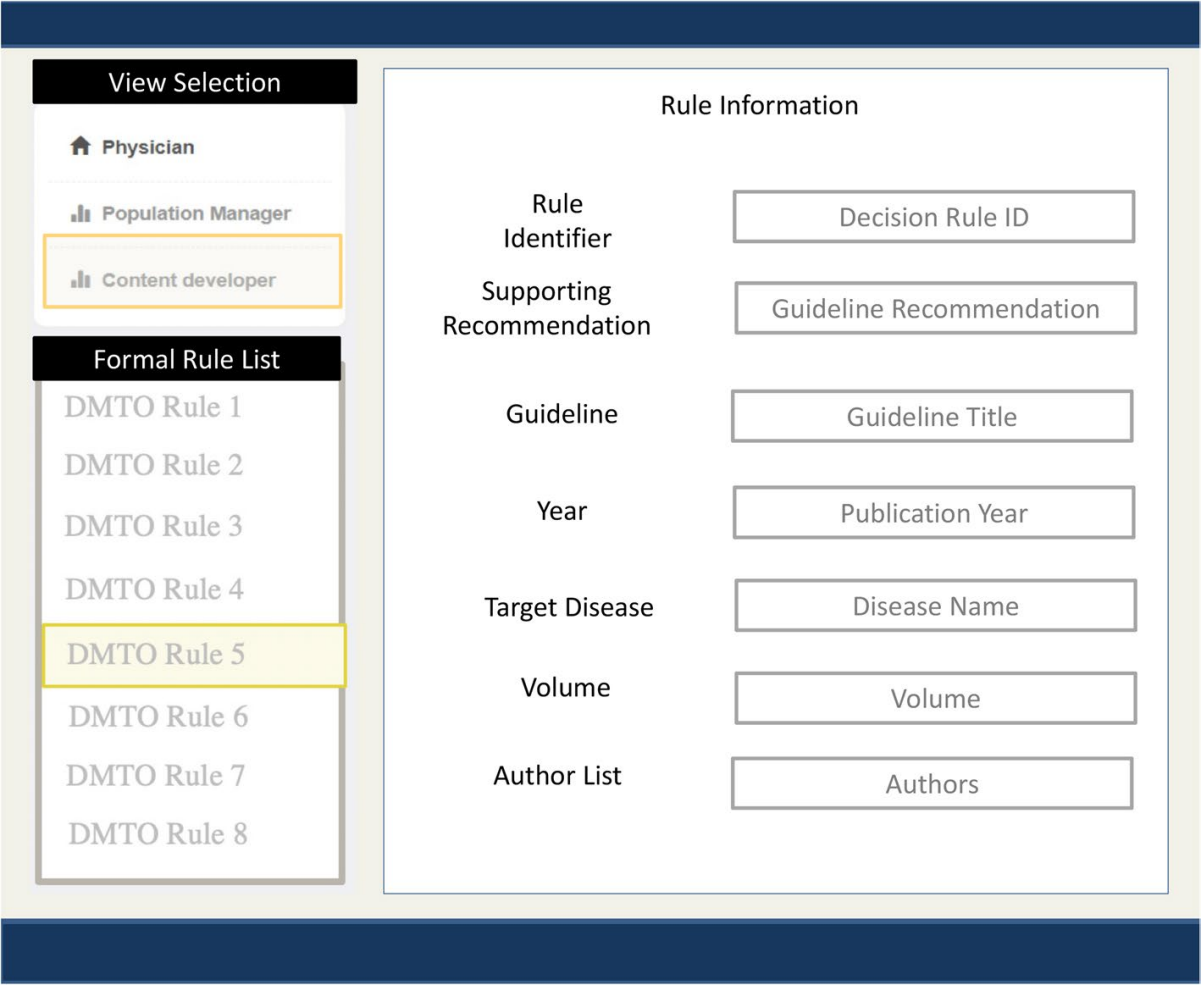
G-Prov CPG Content Developers’ View, where they can input details for new formal rules that are based off of guideline recommendations

In the *healthcare provider’s view* as seen in Fig. 7, provenance information is displayed alongside the CPG recommendation. This includes the source of information in the recommendation, other medical literature that supports the recommendation, and the publication date of the recommendation. Thus, when a CDS system makes a treatment suggestion, the healthcare provider will be able to query the system for more information. In the *content developer’s view* as seen in Fig. 8, there is a form to collect information from the user in a friendly, easy to read manner to create the RDF snippet of the information entered using the G-Prov ontology. Compared to other editing tools such as Protégé, the added advantage of this is that this view does not assume that the user has any semantic web technology knowledge. In the *population health manager’s view* as seen in Fig. 10, we have incorporated the Study Cohort Ontology, which is described in detail in Section Modeling of Study Cohorts in Research Studies Cited in Clinical Practice Guidelines. Using this view, it is possible to analyze the research publications linked from the guidelines, which serve as evidence for recommendations to find any relevant research publications for a patient cohort.

#### Explaining CDS Decisions

Providing coherent explanations in CDS systems is another core objective of our work in addition to representing the provenance of guidelines, which provides one dimension of explanations. Even though CPGs enable evidence-based, clinical decision making, observed clinical actions could deviate from recommended actions. Therefore, it is equally essential to be able to explain the rationale behind such treatment decisions. Thus, there is a need for novel reasoning strategies that provide explanations when clinical decisions do not align with the guidelines. To address this challenge, we introduced a taxonomy of literature-derived explanation types with refined definitions of nine explanation types in [97]. Using that initial taxonomy, we created an Explanation Ontology (EO) for clinical settings [98]. The explanation types in the EO include:

$$Case Based -$$
“To what other situations has this recommendation been applied?”
$$Contextual -$$
“What broader information about the current situation prompted the suggestion of this recommendation?”
$$Contrastive -$$
“Why choose option A over option B that I typically choose?”
$$Counterfactual -$$
“What if input A was B instead?
$$Everyday -$$
“Why does option A make sense?”
$$Scientific -$$
“What research publications have backed this recommendation?”
$$Simulation Based -$$
“What would happen if this recommendation is followed?”
$$Statistical -$$
“What percentage of people with this condition have recovered?”
$$Trace Based -$$
“What steps were taken by the system to generate this recommendation?”

To elicit EO’s application requirements, we conducted a user study in the clinical domain [99]. The study results indicated that healthcare providers most often used or required *contrastive*, *counterfactual*, and *contextual* explanations to understand and reason about complicated patient cases. However, they indicated the need for diverse explanation types to address a range of questions. Therefore, in deriving such semantics-driven explanations to assist healthcare providers, we move closer towards creating more intelligent CDS systems that can perform clinical diagnostics with the use of the EO in conjunction with G-Prov.

#### Software Availability

G-Prov website at [100] contains comprehensive documentation of the ontology and the applications discussed. Utilizing G-Prov, KGs similar to the ADA guideline provenance we created can be created for other diseases. The Explanation Ontology that can be used to encode the criteria useful to explain guideline recommendations is available at [101].

### Representing Study Populations

#### The SCO

The SCO was developed to model the overall structure and patterns of cohort variables and control/intervention groups defined within the structured population descriptions (commonly referred to as *Table1’s* or *Cohort Summary Tables*) of observational case studies and RCTs. We maintain only domain agnostic and commonly accruing subject variables and interventions in SCO, and we have a suite of ontologies (*Diseases*, *Medications*, *LabResults* etc.,) to encode Diabetes related terminology. We have adopted a bottom-up approach to modeling and we have revised the structure of SCO upon investigation of several research studies from the “Pharmacological Interventions” and “Cardiovascular Complications” of the 2018 ADA CPG [94]. The ontology contains 3,568 classes and 629 properties. It is available for download and reuse at [102].

#### Study Cohort KG

**Fig. 9:**
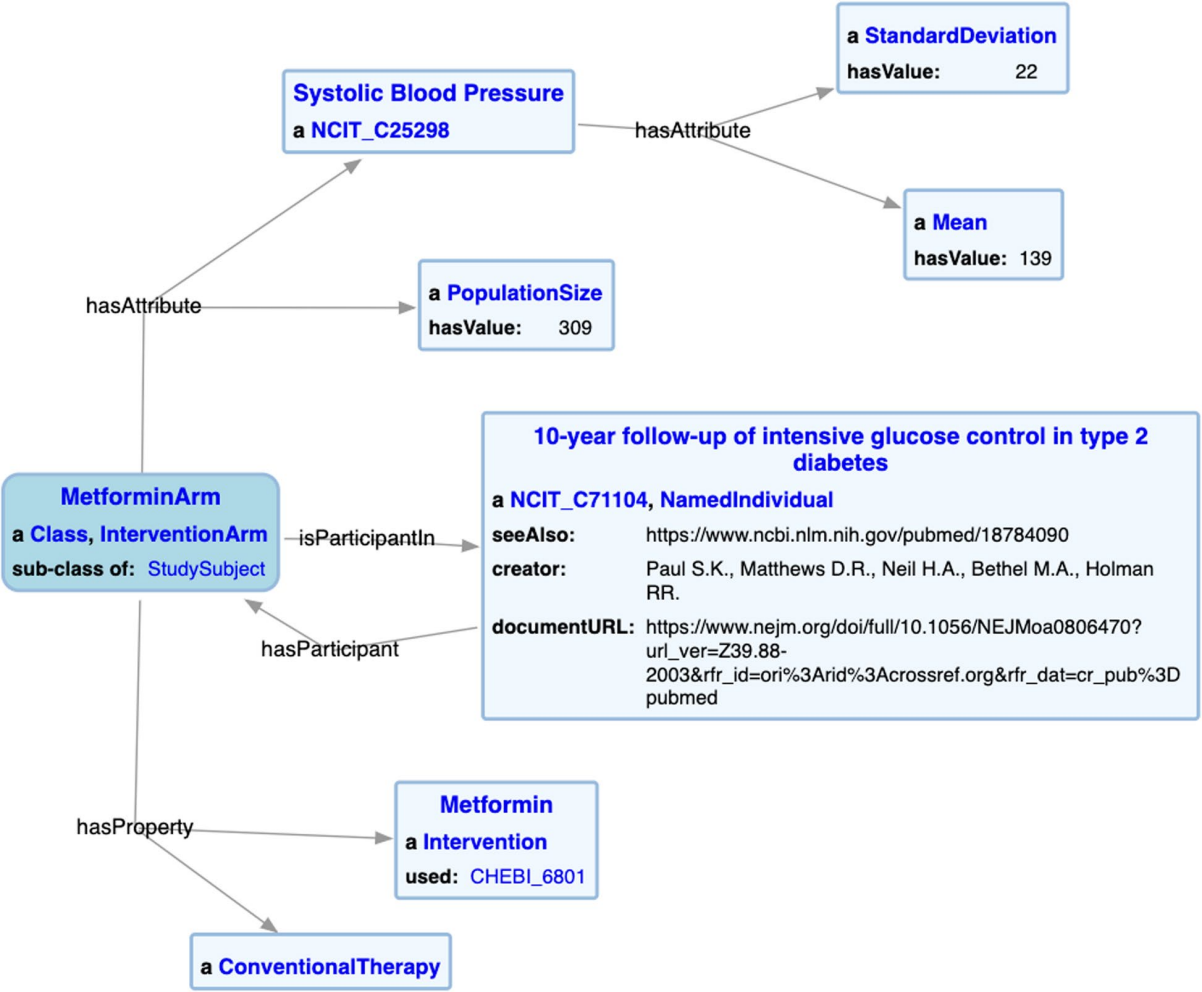
SCO KG snippet

As a proof-of-concept, we modeled 41 cited research publications in the pharmacologic recommendations and cardiovascular complications chapters in the ADA CPG. 19 of these publications came from the 2018 ADA CPG [94], and 22 came from the 2019 ADA CPG [103]. Furthermore, for Medline citations, we have extracted additional study metadata from PubMed and plan to incorporate mappings to Medical Subject Heading (MeSH) terms. Figure 9 highlights the modeling of the fundamental components in SCO, including the study subject collection, their subject characteristics, and the statistical measures associated with the recorded characteristic.

#### Analyzing Study Populations

Applications may use SCO to support analyses that require a deep understanding of study populations. Providers could choose a subset of characteristics they wish to view as a part of the deep dive, or our visualizations could build off characteristics common to both patients and patient groups studied.

Lenert et al. [104] have developed a couple of compelling visualizations of cohort similarity to county populations. However, we did not find sufficiently detailed descriptions about their data analysis, knowledge representation, and machine learning methods to support our direct reuse. Additionally, study bias is common in scientific research [105] and we did not find direct evidence that previous approaches provided enough transparency to support identification of bias or confirmation of no bias. Later in this section, we demonstrate how our knowledge representation of population descriptions can uncover some of these biases.

**Fig. 10:**
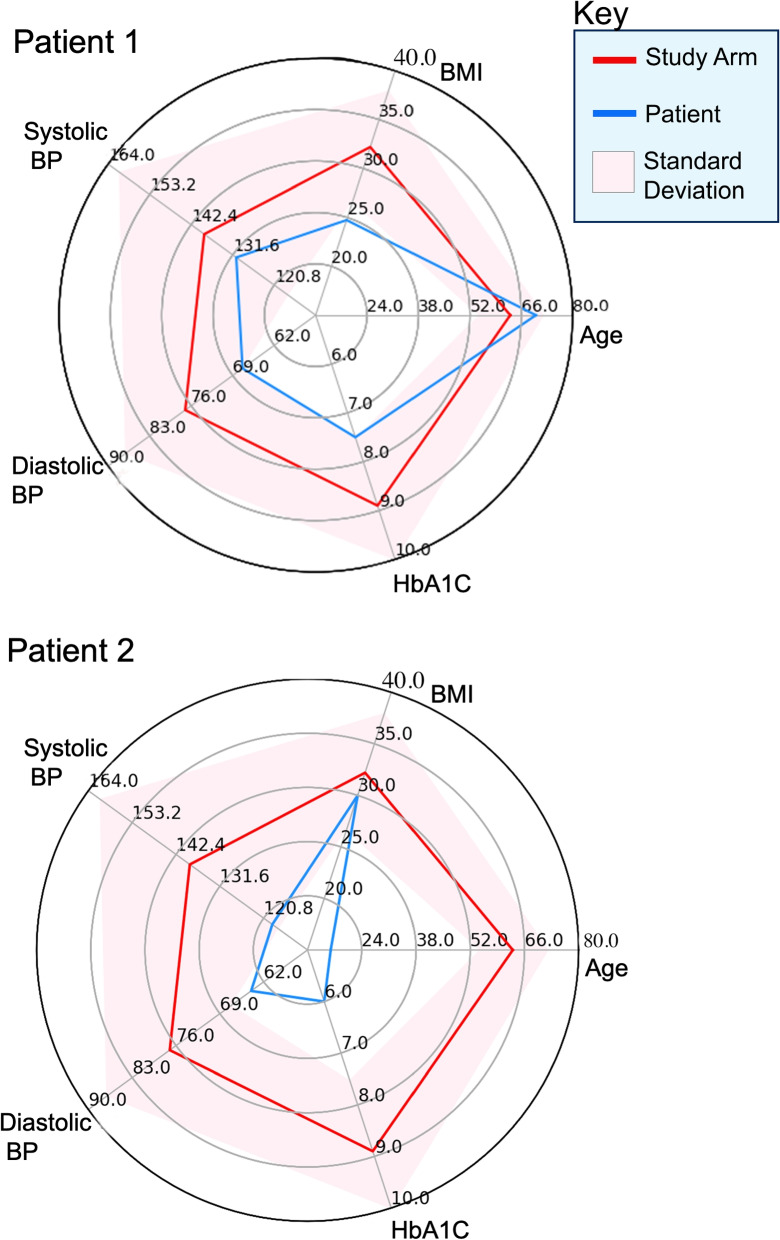
Cohort Similarity Application using SCO and GProv

For an example SCO application, consider the star plots in Fig. 10, which overlay a set of characteristics of the *Metformin study arm* from the “10-year follow-up of intensive glucose control in type 2 diabetes” study cited in chapter 8 of the ADA CPG [94]. The study characteristics are displayed in red, and patient characteristics are displayed in blue. For this illustration, a couple of patients exemplifying a good study fit and a bad study fit are selected from the National Health And Nutrition Examinations 2015-2016 Survey (NHANES) [106]. It is clear that *patient 1* is an excellent match to the study, because on almost all axes, i.e., the age, diastolic blood pressure, hemoglobin A1C, etc., the patient is within the mean+/-standard deviation of the values for the corresponding features of the study population. However, for *patient 2*, we see that the patient’s age is below the lowest age range of the study population. Hence from this plot, a healthcare provider can quickly tell that this study does not apply to *patient 2*.

#### Identifying Gaps in Clinical Populations

Our SCO KGs provide the ability to identify gaps in clinical populations utilized by research publications that are cited in CPGs, which are in turn used in CDS systems. To achieve this capability, we designed clinically relevant scenarios around the following:

(i) *Study match:* Is there a study that matches this patient on one or more features? (For example, find studies with a representation of female African American study subjects?)
(ii) *Study limitation:* Is there an absence or an under-representation of population groups in this study? (For example, find study arms without female African American above the age of 70?)
(iii) *Study quality evaluation:* Are there adequate population sizes and is there a heterogeneity of treatment effects among arms? (For example, find studies with cohort sizes > 1000 and study arm administered drugs of the guanidines family, with sizes 1/3 of those cohort sizes?)

We addressed a competency question for each of these scenarios (explained in detail in [55]). From the data available in the research publications we considered [107], we found that $75\%$ of them had a representation of African-American patients, $\approx 50 \%$ considered older adults above the age of 70, and only $6 \%$ of the large-scale research publications (total population size across study arms $\ge 1000$) had a good treatment to effect ratio. While we have demonstrated visualization and the gap identification capabilities, we believe that the declarative representation of study cohorts can enable further analyses to further evidence-based medicine.

#### Software Availability

The Study Cohort Ontology website [108] contains comprehensive documentation on our knowledge representation approach backed by our study cohort ontology and the knowledge graphs instantiating research study tables.

## Discussion

### Summary

CDS systems have been viewed as aiding health care providers in diagnosis, treatment, and patient monitoring. We demonstrated knowledge representation and engineering techniques supported by semantic technologies to be very suitable to handle heterogeneous and distributed clinical information and decision criteria available in CPGs. Such semantic technologies support the integration of heterogeneous knowledge, the expression of rich and well-defined models for knowledge aggregation through knowledge acquisition pipelines, and the application of logical reasoning for the generation of new knowledge and clinical decision criteria. Since the representation of knowledge is static in traditional CDS systems, the type of knowledge represented can be limiting, which motivates the adoption of semantically-rich and agile software systems. As described in Section Characterizing Disease Based on Guideline Modeling, we applied the semantic technologies we developed in cases where it is useful to have the relevant information for diagnoses that are highly changeable due to the natural evolution of medical research and where new findings and advances are being continuously made.

Furthermore, we show how semantic technologies can support interoperability of terminologies that are needed when interacting with multiple CDS systems. Using the Semantic Web technologies described in this paper, we achieve high interoperability because we use standards-based, community-accepted vocabularies, and practices. For example, in our G-Prov Ontology, we reused community accepted ontologies including the PROV-O [41] and BIBO [43]. In the SCO [55], we reused other study design ontologies such as ProvCaRe [44], widely utilized medical terminologies such as the NCIT [39], DO [54, 70], statistical ontologies such as the UO [109], and STATO [62], and upper-level ontologies such as the SIO [53].

When developing our ontologies described in this paper (i.e., BCS7, BCS8, G-Prov, and SCO), we adopted a use case and competency question-driven approach [110]. Wherever we needed to import a portion of a large-scale ontology, we utilized community accepted practices such as Minimum Information to Reference an External Ontology Term (MIREOT) [111]. Furthermore, we designed our ontologies to interoperate with each other. For example, the G-Prov ontology can also provide SCO information about the citations for the recommendation, and SCO, in turn, will provide information about the patient cohorts used within the study. Therefore, we can trace back the provenance for cited clinical research publications, and vice versa. Also, in our work, the usage of semantic technologies is not limited to knowledge representation. Where applicable, we include a significant focus on the reuse from the expanding body of biomedical ontologies. The CDS semantic application we developed leverages the Whyis knowledge graph framework, which provides a flexible application ecosystem.

### Limitations

The work described in this paper is a prototype CDS system developed under the guidance of a clinical expert who is also a co-author of the paper. However, further validation of the system’s efficacy in real-world user study settings is necessary. Nevertheless, we believe that integrating interoperable semantic technologies in CDS systems has many benefits. Healthcare providers will be able to navigate CPGs better, apply the most appropriate guidelines to their patients without having to pour over voluminous documentation, utilize advanced reasoning capabilities that provide coherent explanations, and ultimately deliver the best care possible for their patients in an equitable manner.

### Future Work

Using the ontologies we have developed as a springboard, we are investigating novel reasoning mechanisms with explanations. Healthcare providers agree that it may not always be wrong to deviate from a CPG if there are good clinical reasons, such as the context of a patient’s unique situation [23]. Healthcare providers should consider any concomitant disease(s) and underlying risk factors when following the recommendations given in CPGs. Ontologies targeted for CDS systems should capture the providers’ reasoning processes by defining clinical concepts, mapping patient data to these concepts, and defining the semantic relationships. For example, a provider should be able to indicate that a particular medication needs to be prescribed to the patient, even though the patient is on a medication that could potentially interact with the prescribed drug because the patient has previously tolerated the medication combination. A systematic review of clinical research ontologies that analyzed 38 different reasoning systems published in the literature [24] outlines various challenges in reasoning systems, which motivates the need to have such special-purpose reasoning that explains certain clinical decisions. We are investigating mechanisms using the ontologies we have developed to understand which guideline recommendation is applicable in the context of discrepancies between past interventions applied to an individual patient and what the guideline would have recommended at those decision points. Specifically, we are expanding reasoning capabilities in the Whyis KG framework [82] by developing a semantically-enabled special-purpose clinical reasoning component to assist in discovering the potential rationales of past treatment options. This reasoner utilizes special-purpose, hybrid reasoning strategies (i.e., deductive and abductive reasoning capabilities), in conjunction with the ontologies we have developed to address some of the lapses in reasoning systems deployed on CDS systems to guide the providers in their treatment decisions. We plan to evaluate the success of the clinical inference activities powered by semantics by comparing healthcare provider conclusions to that of the system and examining discrepancies between the sets of conclusions. Additionally, we are developing an interactive web-based application founded upon RCT equity metrics [112], SCO, and Artificial Intelligence (AI) techniques to measure inequities caused by unrepresentative uni-/multi-variable subgroups in clinical research publications and guide equitable decisions for diverse users, including healthcare providers, researchers, and health policy advocates. Our semantic tools and techniques, especially the SCO, support the calculation of the equity metrics, which requires an aligned analysis of the patient characteristics, the study data, and reference surveillance data (e.g., NHANES) necessary for the cohort similarity calculations and visualizations.

## Conclusions

In this paper, we highlighted several challenges in encoding guideline-based recommendations of today’s clinical decision support systems, along with our methods for addressing the challenges. There are numerous CPGs for effective diagnosis and treatment of diseases that change rapidly as new evidence for the disease comes to light, and there is a great deal of heterogeneous data of varying quality that healthcare providers may need to consider when making treatment decisions. We demonstrated several technologies that lay the foundation towards semantically enriched CDS systems.

One of our main focuses was to develop a scalable knowledge extraction pipeline using semantic technologies to address the challenges that healthcare providers face when creating and evolving a treatment plan for their patients. As an example, we converted the CPG for breast cancer staging into a computer-interpretable guideline representation with links to their cited research publications. We found that connecting recommendations in guidelines to the source literature and their study populations preserves the provenance and enhances the transparency of the decision rules that could be implemented in CDS systems. We furthermore considered the diabetes guidelines and demonstrated how the information contained in the guideline documentation could be extracted at various levels of specificity to ascertain the provenance of the guidelines and understand cohort characteristics of the research studies contained in the guideline recommendations.

We argue that characterizing diseases using semantic technologies is needed because, with the rapid advancements in medical research, the information useful for characterizing diseases, such as diabetes and cancer, has been changing at a pace that creates challenges when providers try to remain current. We provide an example of the value of our approach using our breast cancer application (Section Characterizing Disease Based on Guideline Modeling) that demonstrates how providers may easily re-stage existing patients or patient populations, allowing them to find patients whose stage has changed within a given patient cohort. Our work also includes guideline provenance for attributing the decision rules to the relevant guideline recommendations where we capture the evolution and application of guidelines in a highly evolving medical information space through G-Prov (Section Modeling of Guideline Provenance). We demonstrated how to utilize the G-Prov encodings for annotating CPG recommendations implemented as computer-interpretable SWRL rules for diabetes and how to discover the study populations these recommendations are based on. Furthermore, we believe that leveraging information available in scientific study cohorts will enable systems that are tailored to the unique needs of health professionals to give personalized recommendations based on their patients’ unique situations (Section Modeling of Study Cohorts in Research Studies Cited in Clinical Practice Guidelines). We demonstrated how the SCO annotated KGs support visualizations that provide the capability to view the fit of a patient as a whole, with the various treatment arms, to help the provider ascertain study applicability. Thus, providers can reliably find information tailored to their patients from CPGs, as well as research publications cited in the CPGs, which is generally a difficult task given the limited time they have to peruse large volumes of literature. Finally, we discussed how the application of the tools and techniques described in this paper could supplement CDS systems with advanced reasoning processes, timely and relevant explanations, and analysis of equity of RCTs the CPGs are based on (Section Discussion).

Based on our discussions with healthcare practitioners [99], we believe that our semantic web-based approach of providing enhanced, evidence-based, clinical knowledge to healthcare providers will make their workstreams much more efficient, and, more importantly, lead to increased trust in clinical decision support recommendations.

## Availability and Requirements

- **Project name:** Health Empowerment by Analytics, Learning, and Semantics (HEALS)
- **Project home page:** https://idea.rpi.edu/research/projects/heals-semantic-resources-cds
- **Operating system(s):** Platform independent
- **Other requirements:** None
- **License:** Apache 2
- **Any restrictions to use by non-academics:** None

## Data Availability

Data and the software for the various use cases described in this paper are available at this aggregated resource page at [113]. There, the reader can find more information about the various projects described in this paper that include: $\bullet$ Breast Cancer Staging Ontologies [86] $\bullet$ Explanation Ontology [101] $\bullet$ Guideline Provenance Ontology [100] $\bullet$ Study Cohort Ontology [108] $\bullet$ Whyis Deductive Inference Agent [87] More information about the overall HEALS project, of which the work described in this paper is a part of, is available at [114]. Any other information is available from the corresponding author on a reasonable request.

## Abbreviations

ADA: American Diabetes Association
AI: Artificial Intelligence
AJCC: American Joint Committee on Cancer
BCS: Breast Cancer Staging
BCS7: Breast Cancer Staging 7$^{th}$ Edition
BCS8: Breast Cancer Staging 8$^{th}$ Edition
BIBO: Bibliographic Ontology
CDS: Clinical Decision Support
CHEAR: Children’s Health Exposure Analysis Resource
CIViC: Clinical Interpretations of Variants in Cancer
CMO: Clinical Measurement Ontology
CPG: Clinical Practice Guideline
CST: Cancer Staging Terms
CTO-NDD: Clinical Trials Ontology - Neuro Degenerative Diseases
DCT: Dublin Core metadata Terms
DMTO: Diabetes Mellitus Treatment Ontology
DOID: Disease Ontology
EO: Explanation Ontology
G-Prov: Guideline Provenance Ontology
HAScO: Human-Aware Science Ontology
HEALS: Health Empowerment by Analytics, Learning, and Semantics
HHEAR: Human Health Exposure Analysis Resource
KG: Knowledge Graph
MeSH: Medical Subject Heading
MIREOT: Minimum Information to Reference an External Ontology Term
NCBO: National Center for Biomedical Ontology
NCCN: National Comprehensive Cancer Network
NCIT: National Cancer Institute Thesaurus
NHANES: National Health And Nutrition Examinations 2015-2016 Survey
OCRe: Ontology of Clinical Research
OCTA: Ontology for Cancer Therapy Application
OWL: Web Ontology Language
PATO: Phenotypic Quality Ontology
PROV-O: Provenance Ontology
ProvCaRe: Provenance for Healthcare and Research
RCT: Randomized Clinical Trial
RDF: Resource Description Framework
SCO: Study Cohort Ontology
SEER: Surveillance, Epidemiology, and End Results
SETL: Semantic, Extract, Transform, and Load
SIO: Semanticscience Integrated Ontology
SNOMED-CT: Systemized Nomenclature of Medicine - Clinical Terms
SPARQL SPARQL: Protocol And RDF Query Language
STATO: Statistical Methods Ontology
SWRL: Semantic Web Rule Language
TNM: Tumor, Node, and Metastasis
UICC: Union for International Cancer Control
UO: Units Ontology
W3C: World Wide Web Consortium

## Footnotes

1 SPARQL is a recursive acronym.
